# An extended exponentiated F distribution with theoretical properties and application to lung survival analysis

**DOI:** 10.1038/s41598-026-47675-4

**Published:** 2026-04-18

**Authors:** Fadal Abdullah Ali Aldhufairi

**Affiliations:** Department of Mathematics, King Khalid University, Abha, 61421 Saudi Arabia

**Keywords:** LTED, LTED-Exp, LTED-Weib, Skewness, Survival, Estimation, Engineering, Mathematics and computing

## Abstract

A new framework for a family of LTED with a free baseline distribution provides efficiency to manage complex data. The induced model outperforms existing distribution models by better capturing data features such as heavy tails, skewness, and multimodality. The development is supported by a rigorous theoretical framework that highlights essential statistical properties and provides robust parameter estimation. Empirical evidence from real data, supported by solid statistical goodness-of-fit tests, validates the practical advantages of the model. The study addresses methodological challenges and lays the groundwork for future research, offering improved statistical analysis and application across various fields.

## Introduction

Flexible probability distributions are crucial in contemporary statistical modeling, especially in areas where data often show characteristics such as heavy tails, skewness, multimodality, or non-standard hazard rate shapes. Such characteristics arise naturally in reliability engineering, biomedical survival studies, climatology, hydrology, actuarial science, and financial risk modeling^1,2^. Classical models, including the exponential, Weibull, gamma, Lomax, and log-logistic distributions, frequently fail to account for these complexities^3^, resulting in biased inferences and suboptimal predictive performance.

To overcome these constraints, extensive research over the last two decades has concentrated on the development of distributional generators. These are transformation-based techniques designed to incorporate a baseline distribution *F* into a more flexible family of distributions. Notable instances include the exponentiated family^4,5^, the beta-generated family^6^, the Kumaraswamy-generated and Kumaraswamy-Marshall-Olkin families^7,8^, and Topp-Leone modifications^9^. Additional generator-based approaches include trigonometric, logarithmic, and composite extensions^10–14^. These generators add shape parameters to enhance the modeling of kurtosis, tail weight, and skewness.

In recent years, numerous investigators have introduced generalized and exponentiated distribution families to improve flexibility in modeling skewness, tail behavior, and hazard-rate shapes. Cordeiro et al. ^15^ presented the exponentiated Weibull-H family, illustrating how extra generator parameters could considerably improve tail behavior and modeling flexibility across various applications. Cordeiro et al. ^16^ established a generalized family that integrates multiple transformation-based distributions into a unified framework. These works highlight the increasing interest in generator-based methods and encourage additional developments to get enhanced and simultaneous control over both body and tail behaviors of probability distributions.

Despite these advances, two important limitations remain unaddressed. Most existing generators rely on a single exponentiation or transformation step, limiting their ability to simultaneously adjust both the body and tail of the distribution. Many competing models show favorable tail flexibility but poor hazard-rate flexibility, or vice versa. Several classical models can capture only restricted hazard shapes, such as monotone or simple bathtub patterns^17,18^. Specifically, most classical one-parameter exponentiation schemes cannot obtain the monotone, unimodal, bathtub, and reversed-bathtub hazard forms.

To address these gaps, we introduce the lambda-tau exponentiated distribution (LTED), a new two-parameter generator defined by a double-exponentiation of the baseline distribution function. Unlike conventional exponentiated-*F* families, which rely solely on the transformation $F(y)^\lambda$, the LTED introduces a second shape parameter $\tau$ applied to the survival function. This two-parameter structure has several benefits. The parameters $\lambda$ and $\tau$ influence the lower and upper tails separately, enabling more accurate modeling of heavy-tailed or sub-exponential data. The LTED can generate monotonic, unimodal, bathtub-shaped, and reversed-bathtub hazard functions, even when the baseline distribution possesses only a monotonic hazard rate. The LTED contains the exponentiated, power-*F*, Marshall-Olkin, and baseline families as special cases, meaning it unifies several widely used distributional structures within a single framework. Closed-form expressions for the PDF, CDF, quantile function, moments, entropy measures, and order statistics can be derived for many standard baselines, making the model computationally practical.

Thus, the LTED contributes new mathematical insight by establishing a generator that simultaneously controls body shape, skewness, and tail behavior through a minimal two-parameter system. From an applied perspective, this flexibility allows the LTED to yield improved goodness-of-fit over classical and recently proposed generators when applied to complex real data.

The main novelty of this work lies in the flexibility achieved by the proposed LTED through the joint use of two shape parameters, which enable separate and simultaneous control of the body and tail behavior of the distribution. This structure yields enhanced hazard-rate flexibility that allows a wide range of hazard behaviors, including monotone and non-monotone shapes, to be accommodated under suitable parameter values, even when the baseline distribution exhibits limited hazard behavior. The theoretical properties and likelihood-based inference procedures are systematically developed, and the practical relevance of the LTED is further shown by its use in practice on real-world data sets.

The remainder of the paper is organized as follows. Section “The Lambda-Tau exponentiated distribution” introduces the LTED and its general formulation. Section “Properties of the LTED” presents its statistical properties. Section “Moments and generating functions” describes several special cases and connections to known distributions. Section “Other cases of the LTED family” presents the results of simulation studies and analyzes real data. Section “Methods of estimation” offers conclusions and outlines future research directions.

## The Lambda-Tau exponentiated distribution

This section introduces the LTED, a new two-parameter distribution generator designed to enhance the flexibility of classical lifetime distributions. The LTED extends the exponentiated-*F* (Exp-*F*) family by integrating a two-stage exponentiation mechanism. Reliability analysis and survival studies commonly encounter skewed and heavy-tailed data, which this extension makes particularly suitable for modeling.

The classical Exp-*F* family transforms a baseline cumulative distribution function (CDF) *F*(*y*) by applying a shape parameter $\lambda > 0$, yielding

1 $$\begin{aligned} F_{\text {Exp-{ F}}}(y) = F(y)^{\lambda }. \end{aligned}$$

In Eq. (1), the exponent $\lambda$ adjusts the skewness and tail behavior of the resulting distribution. The baseline distribution is recovered as a special case when $\lambda = 1$.

### Definition

The LTED introduces an additional shape parameter $\tau > 0$ and applies a second exponentiation to the survival function, resulting in the CDF.

2 $$\begin{aligned} F_{\text {LTED}}(y; \lambda , \tau ) = 1 - \left( 1 - F(y)^\lambda \right) ^\tau , \quad y \in \mathcal {S} \subseteq \mathbb {R}_+, \end{aligned}$$

where $\lambda$ and $\tau$ are both positive.

From a modeling perspective, the LTED framework divides the roles of the two shape parameters. $\lambda$ controls skewness and tail weight by exponentiating the baseline CDF, while $\tau$ adjusts the survival function for greater hazard rate flexibility.

The corresponding probability density function (PDF) is obtained by differentiating Eq. (2) with respect to *y*:

3 $$\begin{aligned} f_{\text {LTED}}(y; \lambda , \tau ) = \lambda \tau f(y) F(y)^{\lambda - 1} \left( 1 - F(y)^\lambda \right) ^{\tau - 1}, \end{aligned}$$

where *f*(*y*) is the PDF associated with the baseline distribution *F*(*y*).

The LTED includes several well-known models that serve as special cases:

1. $\lambda = \tau = 1$, the LTED indicates the baseline distribution. For example, when using the exponential baseline, the LTED returns to the exponential distribution.
2. If $\tau = 1$, the LTED transforms into the classical Exp-*F* distribution.
3. If $\lambda = 1$, the LTED becomes the power-*F* (or Beta-type I-*F*) family,
  $$\begin{aligned} F_{\text {LTED}}(y; 1, \tau ) = 1 - \left( 1 - F(y)\right) ^\tau = 1 - \bar{F}(y)^\tau , \end{aligned}$$
  where $\bar{F}(y) = 1 - F(y)$ is the survival function.

The nested exponentiation mechanism allows LTED to generalize several important distributions and provides a wide range of shapes and hazard rate behaviors. This flexibility enables simple baseline distributions to model complex, skewed, or heavy-tailed phenomena, while maintaining mathematical tractability and computational efficiency.

### Stochastic representation

The LTED admits a useful stochastic representation via beta-distributed transformations. Let $U = F(Y) \sim \text {Uniform}(0,1)$, and define $W = U^\lambda$. Then *W* follows a Beta$(1, \tau )$ distribution with CDF

$$\begin{aligned} \Pr (W \le w) = 1 - (1 - w)^\tau . \end{aligned}$$

The inverse transformation gives

$$\begin{aligned} U = W^{1/\lambda }, \quad Y = Q(W^{1/\lambda }), \end{aligned}$$

where $Q(u) = F^{-1}(u)$ is the quantile function of the baseline distribution. This representation facilitates simulation, estimation, and theoretical analysis.

The following sections examine the statistical properties of the LTED, with a focus on its moments, hazard rate behavior, simulation performance, and applications to real survival data. This exploration highlights the practical flexibility of this new family of distributions.

## Properties of the LTED

The properties derived in this section serve two purposes. They establish the theoretical validity of the LTED family and provide practical tools for statistical inference and computation. To this end, we explore extensions of the PDF, closed-form expressions or integral formulations for moments and generating functions, entropy, quantiles, and the behavior of order statistics. Series expansions and stochastic representations further facilitate numerical evaluation, simulation, and estimation when closed-form expressions are unavailable, enhancing the capacity of the LTED model for adaptation to real-world data.

### Series expansion of the PDF

The series expansion of the PDF is a useful tool to examine how it behaves, especially when direct computation is difficult to understand. The PDF in Eq. (3) can be converted into a representation of an infinite series by using the generalized binomial series. This form of the PDF presents us an insight into the underlying statistical properties. This expansion not only shows how important the CDF is, but it also shows how parameters $\lambda$ and $\tau$ shape the distribution. The PDF for the LTED can be written as an expansion of the series in the following theorem.

#### Theorem 1.1

(Power Series Expansion) The PDF of the LTED can be expressed as an expansion of the series:

$$\begin{aligned} f_{\text {LTED}}(y) = \lambda \tau f(y) \sum _{k=0}^{\infty } (-1)^k \left( {\begin{array}{c}\tau - 1\\ k\end{array}}\right) F(y)^{\lambda (k+1) -1}. \end{aligned}$$

#### Proof

Following Eq. (3), the LTED is of the form

4 $$\begin{aligned} f_{\text {LTED}}(y) = \lambda \tau f(y) F(y)^{\lambda - 1} \left( 1 - F(y)^\lambda \right) ^{\tau - 1}. \end{aligned}$$

Now, the term $(1 - F(y)^\lambda )^{\tau - 1}$ is now being addressed in the binomial expansion. By applying the generalized binomial series, one can conclude that

5 $$\begin{aligned} (1 - z)^r = \sum _{k=0}^{\infty } (-1)^k \left( {\begin{array}{c}r\\ k\end{array}}\right) z^k, \quad \text {for } |z| < 1. \end{aligned}$$

With $z = F(y)^\lambda$ and $r = \tau - 1$, the following is obtained from Eq. (5) as

6 $$\begin{aligned} (1 - F(y)^\lambda )^{\tau - 1} = \sum _{k=0}^{\infty } (-1)^k \left( {\begin{array}{c}\tau - 1\\ k\end{array}}\right) F(y)^{\lambda k}. \end{aligned}$$

Substituting Eq. (6) into Eq. (4), it yields the following

$$\begin{aligned} f_{\text {LTED}}(y)= & \lambda \tau f(y) F(y)^{\lambda - 1} \sum _{k=0}^{\infty } (-1)^k \left( {\begin{array}{c}\tau - 1\\ k\end{array}}\right) F(y)^{\lambda k}\\= & \lambda \tau f(y) \sum _{k=0}^{\infty } (-1)^k \left( {\begin{array}{c}\tau - 1\\ k\end{array}}\right) F(y)^{\lambda (k + 1) - 1}. \end{aligned}$$

$$\square$$

### Survival and hazard functions

Survival and hazard functions play an important role in survival analysis that provides information on the timing of events. These functions assist in identifying the risk variables associated with different outcomes and forecasting future events using past data.

#### Corollary 1.2

The survival function of the LTED is given by

7 $$\begin{aligned} \bar{F}_{\text {LTED}}(y) = \left( 1 - F(y)^\lambda \right) ^\tau , \end{aligned}$$

where $\lambda > 0$ and $\tau > 0$ are shape parameters controlling the tails and hazard rate of the distribution.

#### Theorem 1.3

The hazard rate function (HRF) of the LTED is given by

8 $$\begin{aligned} h_{\text {LTED}}(y) = \frac{\lambda \tau f(y) F(y)^{\lambda - 1}}{1 - F(y)^\lambda }. \end{aligned}$$

This function (8) represents the instantaneous risk of event occurrence at time *y*, given survival up to *y*.

#### Proof

The hazard function is defined as follows:

$$\begin{aligned} h_{\text {LTED}}(y) = \frac{f_{\text {LTED}}(y)}{1-F_{\text {LTED}}(y)} = \frac{f_{\text {LTED}}(y)}{\bar{F}_{\text {LTED}}(y)}. \end{aligned}$$

Then, by applying Eqs. (3) and (7), it follows that

$$\begin{aligned} h_{\text {LTED}}(y) = \frac{\lambda \tau f(y) F(y)^{\lambda - 1} \left( 1 - F(y)^\lambda \right) ^{\tau - 1}}{\left( 1 - F(y)^\lambda \right) ^\tau } = \frac{\lambda \tau f(y) F(y)^{\lambda - 1}}{1 - F(y)^\lambda }, \end{aligned}$$

which shows the required result. $\square$

We show that the LTED hazard rate can be expressed in a closed form by substituting the LTED PDF and survival function, thereby clarifying the influence of the shape parameters $\lambda$ and $\tau$ on hazard behavior. It is important to emphasize that the flexibility of the LTED hazard rate depends on both the generator parameters $(\lambda ,\tau )$ and the choice of the baseline distribution. While the LTED framework can generally produce a wide range of hazard rate shapes, in particular, if the baseline distribution has a strongly monotone hazard structure, such as the Weibull distribution with shape parameter $\beta >1$, the resulting LTED hazard function may remain monotone increasing. In these scenarios, the main advantage of the LTED framework is its ability to enhance tail behavior and perform scale modulation, rather than changing the inherent shape of the hazard function.

### Cumulative hazard and reverse hazard functions

Cumulative and reverse hazard functions play a role in survival analysis and reliability engineering because they enable the evaluation of failure risk over time. The cumulative hazard function *H*(*t*) is defined as the integrated hazard rate over time *t*. This function presents a measure of the cumulative risk over time. Particularly, it is useful for assessing failure in systems with time-dependent risks, such as those affected by aging or external factors.

#### Theorem 1.4

The cumulative hazard function (CHF) of the LTED is given by

$$\begin{aligned} H_{\text {LTED}}(y) = -\tau \log \left( 1 - F(y)^\lambda \right) , \end{aligned}$$

which provides a measure of the cumulative risk of an event over time.

#### Proof

The cumulative hazard function can be expressed as follows:

9 $$\begin{aligned} H_{\text {LTED}}(y) = -\log \bar{F}_{\text {LTED}}(y). \end{aligned}$$

By substituting Eq. (7) into Eq. (9), we derive the following:

$$\begin{aligned} H_{\text {LTED}}(y) = -\log \left( 1 - F(y)^\lambda \right) ^\tau = -\tau \log \left( 1 - F(y)^\lambda \right) . \end{aligned}$$

$$\square$$

The reverse hazard function *R*(*t*) measures the conditional risk that an event has already occurred prior to time *y*. It emphasizes the probability of early events or failures and is particularly useful in reliability engineering, finance, and healthcare decisions. Providing this function in closed form assists in computing probabilities associated with such events, complementing the standard hazard analysis. The parameters $\lambda$ and $\tau$ in the LTED affect the contribution of the baseline cumulative distribution function (CDF) to the shape of the reverse hazard function.

#### Theorem 1.5

The reverse hazard rate function (RHRF) of the LTED is given by

$$\begin{aligned} R_{\text {LTED}}(y) = \frac{\lambda \tau f(y) F(y)^{\lambda - 1} \left( 1 - F(y)^\lambda \right) ^{\tau - 1}}{1 - \left( 1 - F(y)^\lambda \right) ^\tau }. \end{aligned}$$

#### Proof

Following Eqs. (3) and (2), The reverse hazard function, denoted as $R_{\text {LTED}}(y)$, is computed as follows:

$$\begin{aligned} R_{\text {LTED}}(y) = \frac{f_{\text {LTED}}(y)}{F_{\text {LTED}}(y)} = \frac{\lambda \tau f(y) F(y)^{\lambda - 1} \left( 1 - F(y)^\lambda \right) ^{\tau - 1}}{1 - \left( 1 - F(y)^\lambda \right) ^\tau }. \end{aligned}$$

$$\square$$

### Quantile function

The quantile function, also referred to as the inverse cumulative distribution function, is one of the most important concepts for characterizing the distribution of a random variable. Random sampling, simulation studies, and reliability and finance risk assessment benefit from it. The LTED framework indicates that by adjusting the quantile mapping parameters $\lambda$ and $\tau$, one can achieve a more flexible modeling approach for extreme quantiles and tail behavior compared to the baseline distribution.

The quantile function *Q*(*p*) for a random variable *X* with a cumulative distribution function $F(x) = P(X \le x)$ is defined as follows:

$$\begin{aligned} Q(p) = \inf \{ x \in \mathbb {R} : p \le F(x) \} \text { for a probability } p \in [0, 1]. \end{aligned}$$

This feature is particularly advantageous in several applications, including simulation and risk assessment.

#### Theorem 1.6

Let $F^{-1}$ denote the quantile function of the baseline distribution. Then the quantile function of the LTED distribution is given by

$$\begin{aligned} Q(u) = F^{-1} \left( \left[ 1 - (1 - u)^{1/\tau } \right] ^{1/\lambda } \right) , \quad 0< u < 1. \end{aligned}$$

#### Proof

From Eq. (2), let $u = F_{\text {LTED}}(y)$. Then, by solving for $F(y)$, we proceed as follows:

10 $$\begin{aligned} F(y) = \left[ 1 - (1 - u)^{1/\tau } \right] ^{1/\lambda }. \end{aligned}$$

Now, applying the inverse of the baseline CDF in Eq. (10), we obtain the quantile function.

$$\begin{aligned} Q(u) = F^{-1} \left( \left[ 1 - (1 - u)^{1/\tau } \right] ^{1/\lambda } \right) . \end{aligned}$$

$$\square$$

## Moments and generating functions

Moments are statistical measures that present the picture of a probability distribution, such as its center, dispersion, and shape. The first moment, which defines the mean, is one of the most important measures of the central tendency. The second central moment, which defines variance, measures the spread of the data set around its mean. The third moment provides skewness that captures asymmetry, and the fourth moment is known as kurtosis, which describes tail weight and peakedness. Note that each moment contributes to providing a certain characteristic of the distribution.

### Moments of the LTED

We begin with the derivation of the raw moments, which form the basis for calculating all higher-order moments and for linking to moment-generating functions.

#### Theorem 1.7

Let $Y \sim \text {LTED}(\lambda , \tau )$. Then the $r^{\hbox {th}}$ raw moment of $Y$ is given by

$$\begin{aligned} \mu '_r = \mathbb {E}[Y^r] = \lambda \tau \int _0^\infty y^r f(y) F(y)^{\lambda - 1} \left( 1 - F(y)^\lambda \right) ^{\tau - 1} \, dy. \end{aligned}$$

#### Proof

The $r^{\hbox {th}}$ raw moment is defined as:

$$\begin{aligned} \mu '_r = \mathbb {E}[Y^r] = \int _0^\infty y^r f_{\text {LTED}}(y) \, dy. \end{aligned}$$

Substituting the PDF in Eq. (3) produces

11 $$\begin{aligned} \mu '_r&= \int _0^\infty y^r \left[ \lambda \tau f(y) F(y)^{\lambda - 1} \left( 1 - F(y)^\lambda \right) ^{\tau - 1}\right] \, dy \nonumber \\&= \lambda \tau \int _0^\infty y^r f(y) F(y)^{\lambda - 1} \left( 1 - F(y)^\lambda \right) ^{\tau - 1} \, dy, \end{aligned}$$

where *f*(*y*) and *F*(*y*) are the PDF and CDF of the baseline distribution, respectively $\square$

Note that Eq. (11) can be expressed in quantile representation by changing the variable to $w = F(y)^{\lambda }$. After a simple algebraic technique, the following is obtained:

$$\begin{aligned} \mu '_r = \mathbb {E}[Y^r] = \mathbb {E}\big [Q(W^{1/\lambda })^r\big ] = \int _0^1 Q(w^{1/\lambda })^r f_W(w) dw, \end{aligned}$$

where $f_W(w) = \tau (1-w)^{\tau -1}$ corresponds to the density function of a beta distribution with shape parameters $(1, \tau )$.

#### Corollary 1.8

The mean of the LTED is the first raw moment given by

$$\begin{aligned} \mu = \mu '_1 = \lambda \tau \int _0^\infty y f(y) F(y)^{\lambda - 1} \left( 1 - F(y)^\lambda \right) ^{\tau - 1} \, dy. \end{aligned}$$

#### Lemma 1.9

Let $Y \sim \text {LTED}(\lambda , \tau )$. The $n^{\hbox {th}}$ central moment of the LTED is given by

$$\begin{aligned} \mu _n = \mathbb {E}[(Y - \mu )^n] = \sum _{r = 0}^{n} \left( {\begin{array}{c}n\\ r\end{array}}\right) (-\mu )^{n - r} \lambda \tau \int _0^\infty y^r f(y) F(y)^{\lambda - 1} (1 - F(y)^\lambda )^{\tau - 1} dy, \end{aligned}$$

where $\mu = \mu '_1$ is the mean of the LTED.

#### Proof

By employing the binomial expansion and adhering to the central moment’s definition, we can demonstrate that

12 $$\begin{aligned} \mu _n = \mathbb {E}[(Y - \mu )^n] = \mathbb {E}\left[ \sum _{r=0}^{n} \left( {\begin{array}{c}n\\ r\end{array}}\right) (-\mu )^{n - r} Y^r\right] = \sum _{r=0}^{n} \left( {\begin{array}{c}n\\ r\end{array}}\right) (-\mu )^{n - r} \mathbb {E}[Y^r]. \end{aligned}$$

Substituting the raw moment of the LTED from Eq. (11) into Eq. (12) yields the desired result, thereby completing the proof. $\square$

#### Lemma 1.10

The $s^{\hbox {th}}$ incomplete moment of the LTED, evaluated at a threshold *t*, is

13 $$\begin{aligned} I_s(t) = \lambda \tau \int _0^t y^s f(y) F(y)^{\lambda - 1} (1 - F(y)^\lambda )^{\tau - 1} dy, \end{aligned}$$

where $$0< t < \infty$$.

Incomplete moments, statistical measures that capture partial information about a distribution, are useful in economic and financial data analysis. For example, the Lorenz curve uses incomplete moments to illustrate population income inequality. Bonferroni and Zenga curves employed incomplete moments to demonstrate risk and inequality in a variety of situations^1,13^.

#### Proof

The $s^{\text {th}}$ incomplete moment is defined as

14 $$\begin{aligned} I_s(t) = \mathbb {E}[Y^s \cdot \mathbb {I}_{\{Y \le t\}}] = \int _{0}^{t} y^s f_Y(y) dy, \end{aligned}$$

since *Y* is a positive random variable with support $(0, \infty )$. Substituting the PDF of the LTED stated in Eq. (3) into Eq. (14), It yields the desired expression provided in Eq. (13). $\square$

#### Lemma 1.11

The conditional $s^{\hbox {th}}$ moment of the LTED given that $Y \le t$ is

$$\begin{aligned} \mathbb {E}[Y^s \mid Y \le t] = \frac{\lambda \tau }{F_{LTED}(t)} \int _0^t y^s f(y) F(y)^{\lambda - 1} (1 - F(y)^\lambda )^{\tau - 1} dy. \end{aligned}$$

Conditional moments measure the tail behavior of a distribution and play an important role for evaluating risk. They are utilized in insurance pricing and extreme value theory to model potential losses.

#### Proof

Conditional expectation of a nonnegative random variable is defined by

$$\begin{aligned} \mathbb {E}[Y^s \mid Y \le t] = \frac{1}{\mathbb {P}(Y \le t)} \int _0^t y^s f_Y(y) dy = \frac{I_s(t)}{F_{LTED}(t)}. \end{aligned}$$

This result follows directly from Eqs. (14) and (2).

$$\square$$

### Moment generating functions

The Moment Generating Functions (MGF) provides another way to characterize the distribution and link moments to exponential-family techniques. It also facilitates derivation of cumulants, which describe dispersion, skewness, and tail weight. For the LTED, the MGF shows explicitly how the generator parameters $(\lambda , \tau )$ shape the exponential moment behavior, improving the interpretability of tail and risk characteristics.

#### Lemma 1.12

Let $Y \sim \text {LTED}(\lambda , \tau )$. Then the MGF, if it exists, is given by

$$\begin{aligned} M_Y(t) = \lambda \tau \int _0^\infty e^{ty} f(y) F(y)^{\lambda - 1} \left( 1 - F(y)^\lambda \right) ^{\tau - 1} dy. \end{aligned}$$

#### Proof

The MGF of a random variable *Y* is defined as

15 $$\begin{aligned} M_Y(t) = \mathbb {E}[e^{tY}] = \int _0^\infty e^{ty} h(y) \, dy, \end{aligned}$$

where *h*(*y*) represents the baseline of the PDF. By substituting *h*(*y*) in Eq. (15) with the PDF of the LTED as defined in Eq. (3), it follows that

$$\begin{aligned} M_Y(t) = \lambda \tau \int _0^\infty e^{ty} f(y) F(y)^{\lambda - 1} \left( 1 - F(y)^\lambda \right) ^{\tau - 1} dy, \end{aligned}$$

which show the desired result. $\square$

#### Lemma 1.13

Let $Y \sim \text {LTED}(\lambda , \tau )$. Then, for values of *t* where the MGF converges absolutely, it can be expressed as a power series expansion:

$$\begin{aligned} M_Y(t) = \sum _{r=0}^{\infty } \frac{t^r}{r!} \mu '_r, \end{aligned}$$

where $\mu '_r$ represents the $r$th raw moment of the random variable $Y$, which is defined as:

$$\begin{aligned} \mu '_r = \lambda \tau \int _0^\infty y^r f(y) F(y)^{\lambda - 1} (1 - F(y)^\lambda )^{\tau - 1} dy. \end{aligned}$$

#### Proof

By definition, the moment generating function is $M_Y(t) = \mathbb {E}[e^{tY}]$. Using the Maclaurin series expansion of the exponential function leads to the following::

16 $$\begin{aligned} e^{tY} = \sum _{r=0}^{\infty } \frac{(tY)^r}{r!} = \sum _{r=0}^{\infty } \frac{t^r Y^r}{r!}. \end{aligned}$$

Taking the expectation of both sides in Eq. (16) results in the following expression:

17 $$\begin{aligned} M_Y(t) = \mathbb {E}[e^{tY}] = \mathbb {E}\left[ \sum _{r=0}^{\infty } \frac{t^r Y^r}{r!}\right] . \end{aligned}$$

If $\mathbb {E}\left[ e^{|tY|}\right] < \infty$, then the expectation and the infinite summation in Eq. (17) can be interchanged as follows:

18 $$\begin{aligned} \mathbb {E}\left[ \sum _{r=0}^{\infty } \frac{t^r Y^r}{r!}\right] = \sum _{r=0}^{\infty } \mathbb {E}\left[ \frac{t^r Y^r}{r!}\right] = \sum _{r=0}^{\infty } \frac{t^r}{r!} \mathbb {E}[Y^r] = \sum _{r=0}^{\infty } \frac{t^r}{r!} \mu '_r. \end{aligned}$$

Finally, substituting the raw moment $\mu '_r$ into Eq. (18), as presented in Theorem 1.7, completes the proof.

$$\square$$

### Entropy measure

One way to measure the disorder or uncertainty in a distribution is through Rényi entropy. This metric displays the effect of the parameters $(\lambda , \tau )$ on variability and tail behavior in the LTED. Increased entropy indicates outcomes that are more dispersed or unpredictable. This concept is relevant to fields such as reliability analysis, risk assessment, and information theory.

#### Definition 1.14

Let $a > 0$, $a \ne 1$. The Rényi entropy of the LTED is defined as follows:

19 $$\begin{aligned} \mathcal {I}_a = \frac{1}{1 - a} \log \left( \int _0^\infty \left[ h(y) \right] ^a dy \right) . \end{aligned}$$

#### Theorem 1.15

Let *f*(*y*) and *F*(*y*) be the PDF and CDF of the baseline distribution. The The Rényi entropy of the LTED can be expressed as follows:

$$\begin{aligned} \mathcal {I}_a = \frac{1}{1 - a} \log \left( \lambda ^a \tau ^a \sum _{k = 0}^\infty \left( {\begin{array}{c}a(\tau - 1)\\ k\end{array}}\right) (-1)^k J_k \right) , \end{aligned}$$

where the inner integral is

$$\begin{aligned} J_k = \int _0^\infty f(y)^a F(y)^{a(\lambda - 1) + \lambda k} dy. \end{aligned}$$

#### Proof

From the definition given in Eq. (19) and by raising the PDF of the LTED in Eq. (3) to the power of *a*, the following results

20 $$\begin{aligned} [f_{\text {LTED}}(y)]^a = \lambda ^a \tau ^a f(y)^a F(y)^{a(\lambda - 1)} (1 - F(y)^\lambda )^{a(\tau - 1)}. \end{aligned}$$

Now, we expand $(1 - F(y)^\lambda )^{a(\tau - 1)}$ in Eq. (20) using the generalized binomial series as follows:

21 $$\begin{aligned} (1 - F(y)^\lambda )^{a(\tau - 1)} = \sum _{k = 0}^\infty \left( {\begin{array}{c}a(\tau - 1)\\ k\end{array}}\right) (-1)^k F(y)^{\lambda k}. \end{aligned}$$

By substituting this result in Eq. (21) with Eq. (20) into the integral in Eq. (19), it follows that

$$\begin{aligned} \int _0^\infty [f_{\text {LTED}}(y)]^a dy&= \lambda ^a \tau ^a \int _0^\infty f(y)^a F(y)^{a(\lambda - 1)} \sum _{k = 0}^\infty \left( {\begin{array}{c}a(\tau - 1)\\ k\end{array}}\right) (-1)^k F(y)^{\lambda k} dy \\&= \lambda ^a \tau ^a \sum _{k = 0}^\infty \left( {\begin{array}{c}a(\tau - 1)\\ k\end{array}}\right) (-1)^k \int _0^\infty f(y)^a F(y)^{a(\lambda - 1) + \lambda k} dy. \end{aligned}$$

Hence,

$$\begin{aligned} \mathcal {I}_a = \frac{1}{1 - a} \log \left( \lambda ^a \tau ^a \sum _{k = 0}^\infty \left( {\begin{array}{c}a(\tau - 1)\\ k\end{array}}\right) (-1)^k J_k \right) , \end{aligned}$$

where

$$\begin{aligned} J_k = \int _0^\infty f(y)^a F(y)^{a(\lambda - 1) + \lambda k} dy. \end{aligned}$$

$$\square$$

If the baseline function *F*(*y*) is explicitly expressed in a certain form, the integral ($J_k$) may allow for either a closed-form solution or a numerical approximation.

## Other cases of the LTED family

The LTED is a general family capable of generating various known and novel lifetime distributions. This section introduces practical examples derived by applying the LTED generator to baseline distributions commonly encountered in reliability and survival analysis. The derivations include the resulting PDF, CDF, and hazard functions, followed by discussions on the flexibility and shapes of these models.

### LTED-exponential distribution

Let the baseline distribution be the exponential distribution with rate parameter $\theta > 0$. The baseline CDF and PDF are given by

22 $$\begin{aligned} F(y) = 1 - e^{-\theta y}, \ \quad \ f(y) = \theta e^{-\theta y}, \quad y \ge 0. \end{aligned}$$

By substituting Eq. (22) into the LTED framework, the CDF of the LTED-Exponential (LTED-Exp) distribution is derived as follows:

23 $$\begin{aligned} F_{\text {LTED-Exp}}(y) = 1 - \left[ 1 - \left( 1 - e^{-\theta y} \right) ^{\lambda } \right] ^{\tau }. \end{aligned}$$

This formulation in (23) augments the exponential model by incorporating two shape factors, $\lambda$ and $\tau$, which afford enhanced flexibility in modeling distributional characteristics and tail heaviness while preserving analytical control.

The corresponding PDF is

24 $$\begin{aligned} f_{\text {LTED-Exp}}(y) = \lambda \tau \theta e^{-\theta y} \left( 1 - e^{-\theta y} \right) ^{\lambda - 1} \left[ 1 - \left( 1 - e^{-\theta y} \right) ^{\lambda } \right] ^{\tau - 1}. \end{aligned}$$

The density in (24) shows how the generator parameters reweight the exponential baseline, allowing greater flexibility in modeling early- and late-life behavior compared to the classical exponential distribution.

The hazard function is given by

25 $$\begin{aligned} h_{\text {LTED-Exp}}(y) = \frac{\lambda \tau \theta e^{-\theta y} \left( 1 - e^{-\theta y} \right) ^{\lambda - 1} \left[ 1 - \left( 1 - e^{-\theta y} \right) ^{\lambda } \right] ^{\tau - 1}}{\left[ 1 - \left( 1 - e^{-\theta y} \right) ^{\lambda } \right] ^{\tau }}. \end{aligned}$$

Varying $\lambda$ and $\tau$ allows flexible hazard shapes, unlike the constant hazard of the standard exponential. This model generalizes the exponential distribution by introducing the additional shape parameters $\lambda$ and $\tau$, enabling it to capture a variety of hazard rate behaviors, including constant, increasing, and decreasing patterns. For certain parameter values, non-monotone shapes may also arise.

**Figure 1:**
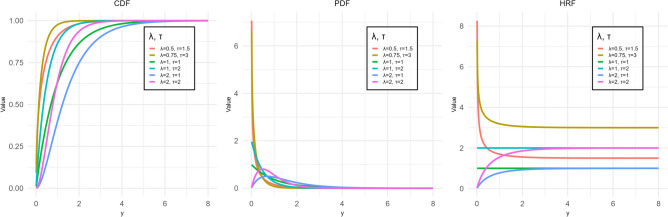
The CDF, PDF, and HRF of the LTED model with an exponential baseline for various combinations of $\lambda$ and $\tau$, with $\theta = 1.25$, illustrating the increased distributional and hazard-rate flexibility relative to the standard exponential model.

Figure 1 depicts the simultaneous impact of altering the shape parameters $\lambda$ and $\tau$ on the distributional framework in relation to the exponential baseline. The CDF and PDF graphs reveal substantial changes in how probability is distributed across time, while the hazard rate function shifts from the constant nature of the exponential model to either increasing or decreasing trends based on parameter values.

For an exponential baseline distribution with rate parameter $\theta > 0$, the quantile function is given by

$$\begin{aligned} Q(u) = -\frac{1}{\theta } \log (1 - u), \quad 0< u < 1. \end{aligned}$$

**Figure 2:**
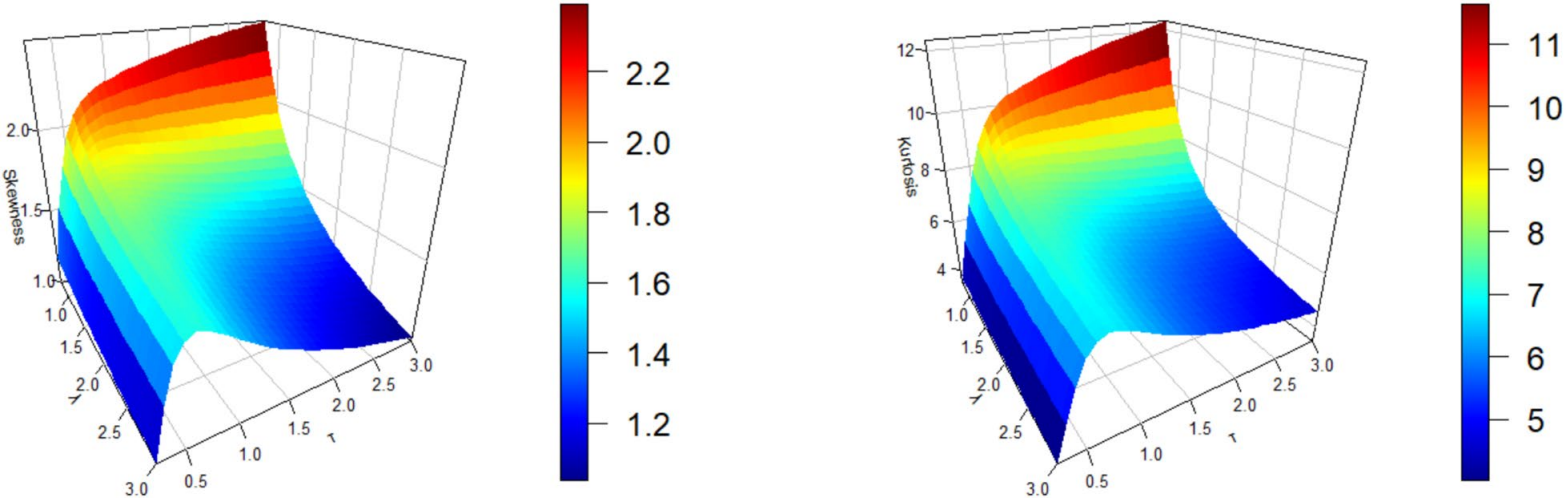
Skewness and kurtosis of the LTED-Exp distribution. The shape parameters $\lambda$ and $\tau$ are varied, while keeping $\theta = 1.25$. The left panel shows the skewness behavior, and the right panel displays the corresponding kurtosis behavior.

Figure 2 shows how $\lambda$ and $\tau$ affect higher-order distributional features. The LTED-Exp model adapts to tail heaviness as shown by the kurtosis surface. The skewness surface indicates that the parameters can either increase or decrease the asymmetry of the distribution.

### LTED-Weibull distribution

Let the baseline distribution be the Weibull distribution with shape parameter $\beta > 0$ and scale parameter $\alpha > 0$. The baseline CDF and PDF are

26 $$\begin{aligned} F(y) = 1 - e^{-(\alpha y)^\beta }, \ \quad \ f(y) = \alpha ^\beta \beta \, y^{\beta - 1} e^{-(\alpha y)^\beta }, \quad y \ge 0. \end{aligned}$$

By substituting Eq. (26) in the LTED framework, the CDF of the LTED-Weibull (LTED-Weib) distribution is derived as follows:

$$\begin{aligned} F_{\text {LTED-Weib}}(y) = 1 - \left[ 1 - \left( 1 - e^{-(\alpha y)^\beta } \right) ^\lambda \right] ^\tau . \end{aligned}$$

The Weibull model is extended through the LTED-Weib distribution, which incorporates shape parameters $\lambda$ and $\tau$. These parameters mostly control tail thickness and scaling while maintaining the Weibull baseline structure. The additional parameters $\lambda$ and $\tau$ increase flexibility in tail behavior and hazard shapes compared to the standard Weibull.

The corresponding PDF is

$$\begin{aligned} f_{\text {LTED-Weib}}(y) = \lambda \tau \, \alpha ^\beta \beta \, y^{\beta - 1} e^{-(\alpha y)^\beta } \left( 1 - e^{-(\alpha y)^\beta } \right) ^{\lambda - 1} \left[ 1 - \left( 1 - e^{-(\alpha y)^\beta } \right) ^\lambda \right] ^{\tau - 1}. \end{aligned}$$

The hazard rate function (HRF) is given by

$$\begin{aligned} h_{\text {LTED-Weib}}(y) = \frac{\lambda \tau \, \alpha ^\beta \beta \, y^{\beta - 1} e^{-(\alpha y)^\beta } \left( 1 - e^{-(\alpha y)^\beta } \right) ^{\lambda - 1} \left[ 1 - \left( 1 - e^{-(\alpha y)^\beta } \right) ^\lambda \right] ^{\tau - 1}}{\left[ 1 - \left( 1 - e^{-(\alpha y)^\beta } \right) ^\lambda \right] ^\tau }. \end{aligned}$$

The behavior of the LTED-Weib hazard function is influenced by both the baseline Weibull shape parameter $\beta$ and the generator parameters $(\lambda ,\tau )$. The LTED improves distributional flexibility, but the hazard rate may remain monotone if the Weibull baseline is monotonic. In such cases, the LTED extension improves tail modeling and fit rather than changing the hazard shape.

**Figure 3:**
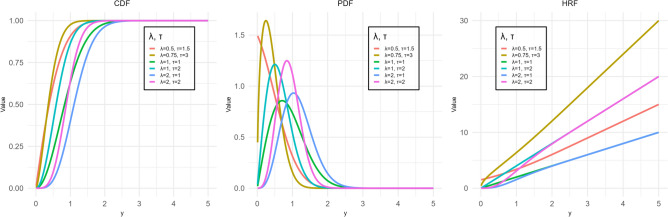
The CDF, PDF, and HRF plots of the LTED model with a Weibull baseline for selected values of $\lambda$ and $\tau$, with $\alpha = 1.25$ and $\beta = 2$. The plots illustrate that, under this baseline value, the hazard rate remains monotone increasing while exhibiting additional tail flexibility.

Figure 3 demonstrates that variations in $\lambda$ and $\tau$ primarily affect tail behavior and the rate at which probability accumulates over time, thereby influencing the magnitude and slope of the hazard rate. These parameters provide greater control over failure dynamics within the LTED framework.

For a Weibull baseline distribution with scale parameter $\alpha > 0$ and shape parameter $\beta > 0$, the quantile function is given by

$$\begin{aligned} Q(u) = \frac{\big (-\log (1 - u)\big )^{1/\beta }}{\alpha }, \quad 0< u < 1. \end{aligned}$$

**Figure 4:**
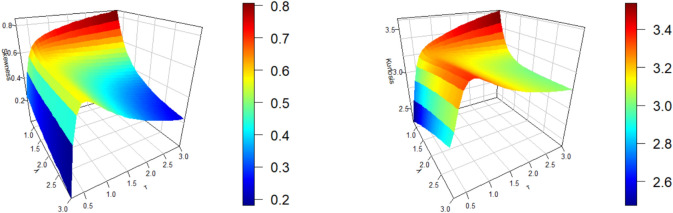
Skewness and kurtosis of the LTED-Weib distribution for varying $\lambda$ and $\tau$ parameters, while keeping $\alpha = 1.25$ and $\beta = 2$. The left panel shows the skewness behavior, and the right panel displays the corresponding kurtosis behavior.

Figure 4 illustrates how the parameters $\lambda$ and $\tau$ affect higher-order characteristics of the LTED-Weib distribution. This distribution effectively captures tail heaviness and asymmetry, as demonstrated by its skewness and kurtosis surfaces. The findings suggest that the LTED-Weib model increases flexibility mainly through adjustments to the tail and moments while maintaining the fundamental structure of the Weibull distribution.

The cases show that the LTED serves as a highly adaptable framework and is applicable for examining a diverse range of different distributions in the fields of survival and reliability analysis. The following sections evaluate the effectiveness of LTED estimators in simulations and their relevance in analyzing real-world applications.

## Methods of estimation

This section offers a detailed analysis of various estimation methodologies relevant to the LTED. The estimation methods considered in this study consist of likelihood-based and minimum-distance estimation. Maximum likelihood estimation (MLE) is included for its asymptotic properties and widespread use in parametric survival modeling. The least squares (LS) and weighted least squares (WLS) estimators are included because they are simple and robust in small samples. The Anderson-Darling (AD) and Cramér-von Mises (CvM) estimators are employed as goodness-of-fit-based methods emphasizing tail behavior for flexible lifetime distributions. This investigation demonstrates the efficacy of the LTED in statistical modeling.

### Maximum likelihood estimation

Let $Y_1,\dots ,Y_n$ be an independent random sample from the LTED distribution with parameter vector $\boldsymbol{\sigma } = (\lambda , \tau , \psi )^\top$, where $\psi$ represents the parameters of the baseline distribution *F*(*y*). The likelihood function for this sample is

27 $$\begin{aligned} L(\boldsymbol{\sigma })&= \prod _{i=1}^n \lambda \tau f(y_i; \psi ) \, F(y_i; \psi )^{\lambda - 1} \left[ 1 - F(y_i; \psi )^\lambda \right] ^{\tau - 1}. \end{aligned}$$

Taking the natural logarithm of both sides of Eq. (27) results in the log-likelihood:

$$\begin{aligned} \ell (\boldsymbol{\sigma })&= n (\log (\lambda ) + \log (\tau ) ) + \sum _{i=1}^n \log f(y_i; \psi ) + (\lambda - 1) \sum _{i=1}^n \log F(y_i; \psi ) \\&\quad + (\tau - 1) \sum _{i=1}^n \log \left[ 1 - F(y_i; \psi )^\lambda \right] . \end{aligned}$$

The MLEs $\widehat{\boldsymbol{\sigma }}$ are obtained by solving the system of likelihood equations:

28 $$\begin{aligned} \frac{\partial \ell (\boldsymbol{\sigma })}{\partial \lambda }&= \frac{n}{\lambda } + \sum _{i=1}^n \log F(y_i; \psi ) - (\tau - 1) \sum _{i=1}^n \frac{F(y_i; \psi )^\lambda \log F(y_i; \psi )}{1 - F(y_i; \psi )^\lambda } = 0, \nonumber \\ \frac{\partial \ell (\boldsymbol{\sigma })}{\partial \tau }&= \frac{n}{\tau } + \sum _{i=1}^n \log \left[ 1 - F(y_i; \psi )^\lambda \right] = 0, \nonumber \\ \frac{\partial \ell (\boldsymbol{\sigma })}{\partial \psi }&= \sum _{i=1}^n \frac{\partial }{\partial \psi } \log f(y_i; \psi ) + (\lambda - 1) \sum _{i=1}^n \frac{\partial }{\partial \psi } \log F(y_i; \psi ) \nonumber \\&\quad - (\tau - 1) \sum _{i=1}^n \frac{\lambda F(y_i; \psi )^{\lambda - 1} }{1 - F(y_i; \psi )^\lambda } \frac{\partial }{\partial \psi } F(y_i; \psi ) = 0. \end{aligned}$$

These equations in (28) are nonlinear in the parameters and typically do not have closed-form solutions. Hence, numerical optimization methods such as Newton-Raphson or BFGS are used to approximate the MLEs $\widehat{\boldsymbol{\sigma }}$. Once obtained, the observed Fisher information matrix

$$\begin{aligned} \mathcal {I}(\widehat{\boldsymbol{\sigma }}) = -\frac{\partial ^2 \ell (\boldsymbol{\sigma })}{\partial \boldsymbol{\sigma } \, \partial \boldsymbol{\sigma }^\top } \bigg |_{\boldsymbol{\sigma } = \widehat{\boldsymbol{\sigma }}}, \end{aligned}$$

can be used to compute standard errors and construct approximate confidence intervals for the estimates.

### Least squares and weighted least squares estimation

Both LS and WLS methodologies can be applied to obtain estimators for the LTED parameters. Let $\boldsymbol{\sigma } = (\lambda , \tau , \psi )^\top$, where $\psi$ denotes the parameters of the baseline distribution $F(y; \psi )$. The LS estimation is formulated as

$$\begin{aligned} \hbox {LS}(\boldsymbol{\sigma }) = \sum _{i=1}^n \left[ F_{\text {LTED}}(y_{(i)}; \boldsymbol{\sigma }) - \frac{i}{n + 1} \right] ^2, \end{aligned}$$

where $y_{(1)} \le y_{(2)} \le \cdots \le y_{(n)}$ denote the ordered sample observations. The WLS estimation, which provides more weight to the tails of the empirical distribution, seeks to minimize

$$\begin{aligned} \hbox {WLS}(\boldsymbol{\sigma }) = \sum _{i=1}^n \frac{(n + 1)^2 (n + 2)}{i (n - i + 1)} \left[ F_{\text {LTED}}(y_{(i)}; \boldsymbol{\sigma }) - \frac{i}{n + 1} \right] ^2. \end{aligned}$$

The LS$(\boldsymbol{\sigma })$ or WLS$(\boldsymbol{\sigma })$ estimators can be obtained by solving the following system of nonlinear equations:

$$\begin{aligned} \frac{\partial Z}{\partial \lambda } = 0, \quad \frac{\partial Z}{\partial \tau } = 0, \quad \frac{\partial Z}{\partial \psi _j} = 0, \quad j = 1, \dots , p, \end{aligned}$$

where *Z* is either $\hbox {LS}(\boldsymbol{\sigma })$ or $\hbox {WLS}(\boldsymbol{\sigma })$. In practice, numerical optimization methods, such as Newton-Raphson or quasi-Newton algorithms, are employed as a result of the absence of closed-form solutions.

### Anderson-darling approach of estimation

The AD method is another powerful tool for parameter estimation. Particularly, it is sensitive to discrepancies in the tails of the distribution. Let $\boldsymbol{\sigma } = (\lambda , \tau , \psi )^\top$ represent the parameter vector of the LTED. The AD criterion is defined as

$$\begin{aligned} \hbox {AD}(\boldsymbol{\sigma })&= -n - \frac{1}{n} \sum _{i=1}^n (2i - 1)\left[ \log F_{\text {LTED}}(y_{(i)}; \boldsymbol{\sigma }) - \log \left( \bar{F}_{\text {LTED}}(y_{(n+1-i)}; \boldsymbol{\sigma }) \right) \right] , \nonumber \\&= -n - \frac{1}{n} \sum _{i=1}^n (2i - 1)\left[ \log \left( 1 - \left[ 1 - F(y_{(i)}; \psi )^\lambda \right] ^\tau \right) - \tau \log \left( 1 - F(y_{(n+1+i)}; \psi )^\lambda \right) \right] . \end{aligned}$$

The AD estimators are obtained by minimizing the function $\hbox {AD}(\boldsymbol{\sigma })$ with respect to $\boldsymbol{\sigma }$, which leads to solving the following nonlinear system of equations:

29 $$\begin{aligned} \frac{\partial \hbox {AD}(\boldsymbol{\sigma })}{\partial \lambda } = 0, \quad \frac{\partial \hbox {AD}(\boldsymbol{\sigma })}{\partial \tau } = 0, \quad \frac{\partial \hbox {AD}(\boldsymbol{\sigma })}{\partial \psi _j} = 0, \quad j = 1, \dots , p. \end{aligned}$$

Equation (29) is frequently accomplished using numerical optimization algorithms such as Newton-Raphson or BFGS methods.

### Cramér-von Mises approach of estimation

The CvM method offers an alternative to maximum likelihood and least squares methods. It is defined by minimizing the squared differences between the empirical and theoretical CDFs across the entire support. The CvM statistic is defined as

$$\begin{aligned} \textrm{CvM}(\boldsymbol{\sigma })&= \frac{1}{12n^2} + \frac{1}{n} \sum _{i=1}^n \left[ F_{\text {LTED}}(y_{(i)}; \boldsymbol{\sigma }) - \frac{2i - 1}{2n} \right] ^2, \nonumber \\&= \frac{1}{12n^2} + \frac{1}{n} \sum _{i=1}^n \left[ 1 - \left( 1 - F(y_{(i)}; \psi )^\lambda \right) ^\tau - \frac{2i - 1}{2n} \right] ^2. \end{aligned}$$

The CvM estimators are obtained by solving

$$\begin{aligned} \frac{\partial \textrm{CvM}(\boldsymbol{\sigma })}{\partial \lambda } = 0, \quad \frac{\partial \textrm{CvM}(\boldsymbol{\sigma }) }{\partial \tau } = 0, \quad \frac{\partial \textrm{CvM}(\boldsymbol{\sigma }) }{\partial \psi _j} = 0, \quad j = 1, \dots , p, \end{aligned}$$

which frequently requires numerical optimization methods.

## Simulation study

A simulation examined LTED estimation methods by comparing two baseline distributions: the exponential distribution, characterized by a shape parameter $\theta$, and the Weibull distribution, which includes both a shape parameter $\beta$ and a scale parameter $\alpha$. The simulation study compares the finite-sample bias and MSE estimation methods in controlled settings.

Each simulation scenario generated random samples from the LTED distribution using the quantile function of the baseline model. In applied survival and reliability studies, sample sizes typically include 50, 100, 250, 500, and 1000. For each sample size and corresponding true parameter values, 1000 independent samples were generated and replicated to ensure the statistical robustness of bias and mean squared error (MSE).

### Simulation of LTED-Exp distribution estimators

The simulations used true parameter values that represented moderate skewness and tail behavior without boundary cases. These values enable realistic estimator performance comparisons without favoring any estimation method.

Table 1 presents the bias and mean square error (MSE) associated with the various methods used to estimate the parameters of the LTED-Exp distribution. The column labeled “Best Est.” indicates the estimation method that achieves the smallest MSE for the corresponding parameter and sample size.

**Table 1:** Bias and MSE of different estimators for the LTED-Exp distribution with $\theta =1/2,$ $\lambda =1/3$, and $\tau =1/2.$.

*n*	Param	Best	MLE		LS		WLS		AD		CvM	
		Est.	Bias	MSE	Bias	MSE	Bias	MSE	Bias	MSE	Bias	MSE
	$\theta$	MLE	0.2150	**0.4422**	0.4665	1.2013	0.3396	0.7702	0.3052	0.6571	0.4885	1.2902
50	$\lambda$	WLS	0.0152	0.0134	-0.0484	0.0160	-0.0323	**0.0114**	-0.0161	0.0116	-0.0305	0.0153
	$\tau$	CvM	0.4939	7.7634	0.2468	1.4026	0.3369	2.3077	0.3933	2.4908	0.2246	**0.9849**
	$\theta$	MLE	0.0995	**0.1819**	0.2033	0.4400	0.1282	0.2768	0.1271	0.2597	0.2183	0.4647
100	$\lambda$	AD	0.0136	0.0100	-0.0175	0.0096	-0.0056	0.0079	-0.0023	**0.0071**	-0.0093	0.0096
	$\tau$	CvM	0.2009	0.7999	0.2293	0.7884	0.2452	0.8422	0.2309	0.8291	0.2266	**0.7650**
	$\theta$	MLE	0.0541	**0.0786**	0.0955	0.1589	0.0602	0.0970	0.0651	0.1053	0.0997	0.1608
250	$\lambda$	AD	0.0108	0.0084	-0.0072	0.0053	-0.0029	0.0037	-0.0024	**0.0036**	-0.0040	0.0052
	$\tau$	MLE	0.0640	**0.0966**	0.1048	0.2491	0.0686	0.1315	0.0642	0.1248	0.1047	0.2522
	$\theta$	WLS	0.0464	0.0469	0.0370	0.0635	0.0210	**0.0384**	0.0285	0.0513	0.0384	0.0648
500	$\lambda$	WLS	0.0069	0.0046	-0.0012	0.0027	0.0005	**0.0017**	0.0001	0.0018	0.0007	0.0027
	$\tau$	MLE	0.0342	**0.0305**	0.0474	0.0552	0.0327	0.0322	0.0302	0.0309	0.0503	0.0579
	$\theta$	WLS	0.0356	0.0287	0.0172	0.0260	0.0102	**0.0169**	0.0318	0.0556	0.0164	0.0266
1000	$\lambda$	WLS	0.0048	0.0018	-0.0013	0.0013	-0.0004	**0.0008**	-0.0022	0.0011	-0.0000	0.0013
	$\tau$	WLS	0.0290	0.0202	0.0196	0.0201	0.0137	**0.0124**	0.0091	0.0135	0.0233	0.0228

**Figure 5:**
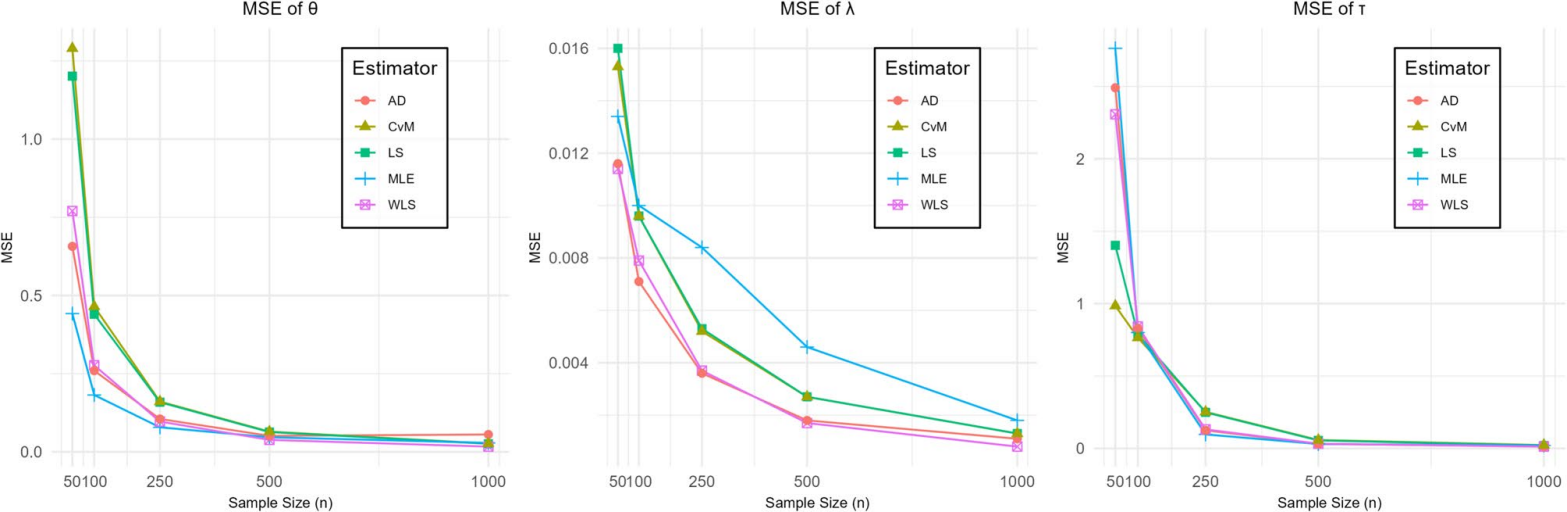
MSE of different estimators across sample sizes for the LTED-Exp model.

The results shown in Table 1 and Fig. 5 indicate that MLE and WLS yield reduced bias and MSE values in the majority of scenarios relative to alternative estimators across various sample sizes.

When the sample size is small, biases are comparatively high but decrease gradually as the sample size increases. Although the LS and AD approaches exhibit higher MSEs and bias in smaller samples, their efficacy enhances with larger samples. The LS estimator displays comparable performance to CvM.

### Simulation of LTED-Weib distribution estimators

An analysis assesses the effectiveness of various estimation methods for the LTED model in finite samples, particularly when the baseline distribution is a Weibull distribution provided in (26). This distribution offers increased flexibility via its shape parameter and scale parameter.

**Figure 6:**
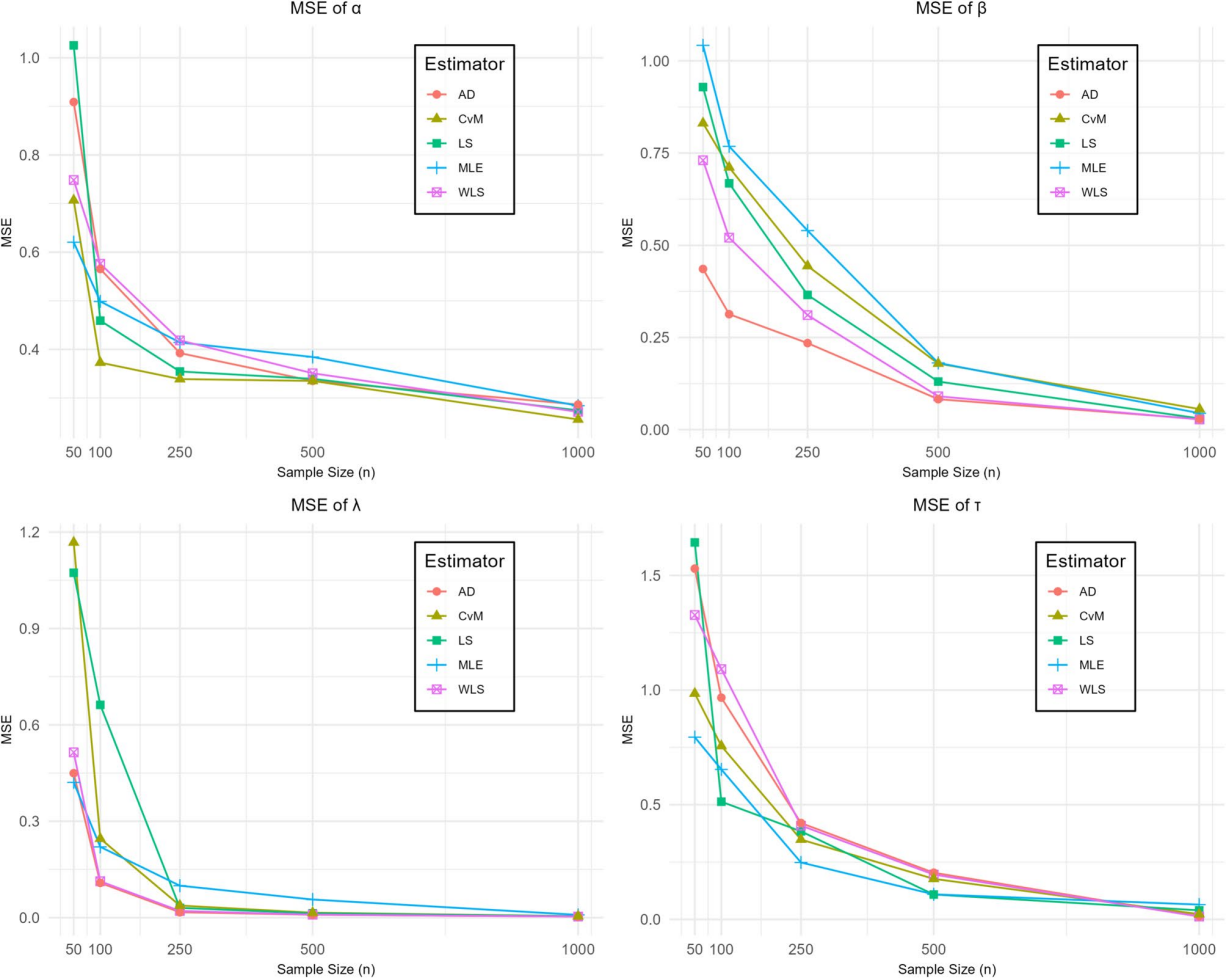
MSE of different estimators across sample sizes for the LTED model with a Weibull baseline.

Unlike the LTED-Exp distribution, Table 2 and Fig. 6 show that the LS, AD, and CvM have reduced bias and MSE values for certain parameters across various sample sizes. The AD and CvM estimates generally show intermediate bias and often have a lower MSE than the LS estimate for moderate to large sample sizes. The results emphasize that no single estimating method is superior. The performance of the estimators varies with both sample size and parameter type, highlighting the benefit of considering multiple estimation approaches.

**Table 2:** Bias and MSE of different estimators for the LTED-Weib distribution with $\alpha =2/3$, $\beta =9/8$, $\lambda =1/2$, and $\tau =1/3$ .

*n*	Param	Best	MLE		LS		WLS		AD		CvM	
		Est.	Bias	MSE	Bias	MSE	Bias	MSE	Bias	MSE	Bias	MSE
50	$\alpha$	MLE	− 0.6484	**0.6205**	0.0770	1.0255	0.0193	0.7484	0.1730	0.9089	− 0.0260	0.7068
	$\beta$	AD	1.2039	1.0421	0.3149	0.9290	0.2875	0.7305	0.2000	**0.4357**	0.2572	0.8311
	$\lambda$	MLE	− 0.4685	**0.4211**	0.2835	1.0732	0.1692	0.5145	0.1788	0.4495	0.3456	1.1676
	$\tau$	MLE	0.5325	**0.7945**	0.6520	1.6434	0.5442	1.3270	0.5757	1.5294	0.2246	0.9849
100	$\alpha$	CvM	− 0.6468	0.4986	0.6407	0.4591	0.4872	0.5761	0.1421	0.5653	− 0.1523	**0.3727**
	$\beta$	AD	1.1478	0.7683	0.3187	0.6680	0.3150	0.5206	0.1498	**0.3131**	0.3251	0.7114
	$\lambda$	AD	− 0.4679	0.2203	0.1009	0.6622	0.0312	0.1133	0.0607	**0.1083**	0.1052	0.2460
	$\tau$	LS	0.2425	0.6539	0.2570	**0.5132**	0.5824	1.0912	0.5330	0.9668	0.5494	0.7560
250	$\alpha$	CvM	− 0.6457	0.4142	− 0.1400	0.3544	0.2589	0.4184	0.2369	0.3923	− 0.1379	**0.3388**
	$\beta$	AD	0.4651	0.5398	0.2394	0.3654	0.2272	0.3106	0.1753	**0.2347**	0.2667	0.4438
	$\lambda$	AD	− 0.0549	0.0995	− 0.0745	0.0304	− 0.0653	0.0209	− 0.0563	**0.0169**	− 0.0606	0.0377
	$\tau$	MLE	0.0984	**0.2481**	0.1722	0.3839	0.2225	0.4090	0.2195	0.4201	0.1839	0.3481
500	$\alpha$	CvM	− 0.4291	0.3842	− 0.1441	0.3393	0.1975	0.3508	0.2306	0.3351	− 0.1355	**0.3350**
	$\beta$	AD	0.1889	0.1810	0.0973	0.1304	0.1056	0.0904	0.0949	**0.0826**	0.1350	0.1792
	$\lambda$	AD	− 0.0515	0.0565	− 0.0518	0.0136	− 0.0369	0.0094	− 0.0364	**0.0091**	− 0.0512	0.0154
	$\tau$	LS	0.0520	0.1093	0.0323	**0.1085**	0.1333	0.1955	0.1115	0.2035	0.0870	0.1768
1000	$\alpha$	MLE	− 0.2289	**0.0445**	− 0.1024	0.2744	0.1364	0.2713	0.1520	0.2864	− 0.1145	0.2557
	$\beta$	WLS	0.8077	0.7245	0.0283	0.0304	0.0296	**0.0276**	0.0344	0.0287	0.0541	0.0557
	$\lambda$	AD	− 0.0307	0.0087	− 0.0234	0.0044	− 0.0197	0.0036	− 0.0186	**0.0035**	− 0.0191	0.0046
	$\tau$	WLS	0.045 9	0.0645	0.0224	0.0395	0.0137	**0.0124**	0.0091	0.0135	0.0233	0.0228

Comparing LTED-Exp and LTED-Weib simulations shows that baseline complexity affects estimator performance. The MLE and WLS methods demonstrate improved bias and MSE as sample size increases in the LTED-Exp model, due to the simpler structure of the exponential baseline. The shape and scale parameters of the LTED-Weib model affect estimator behavior, with the LS, AD, and CvM methods having lower MSE values for certain parameters and sample sizes.

Generally, the simulation results point out that the performance of the estimation improves with an increasing sample size across all five methods. This overall trend supports the practical reliability of the proposed estimation procedures and highlights their suitability for real-world data analysis, thereby strengthening the applicability of the LTED framework for statistical modeling.

## Application to lung survival data

To further evaluate the modeling capability of the LTED family, we apply the LTED-Exp and LTED-Weib distributions to real-world lung survival data. This data used in this study consists of 168 patient records. It was obtained from the Public Medical Datasets for Survival Analysis collection hosted by Virginia Tech. Access to these datasets is available through the Virginia Tech Survival Datasets platform.

### Model comparison for lung survival data

Exponential, Weibull, LTED-Exp, and LTED-Weib models were evaluated to determine the most appropriate parametric survival model for the lung dataset. The assessment of model fit included several criteria, such as the negative twice log-likelihood ($-2\log L$), the Akaike Information Criterion (AIC), the Bayesian Information Criterion (BIC) given as $\text {BIC} = -2 \cdot \text {LogLik} + \log (n) \cdot k,$, the root mean squared error (RMSE), the Kolmogorov-Smirnov (KS) statistic, and the corresponding *p*-values. Here, AIC and BIC are defined as

$$\begin{aligned} \text {AIC} = -2 \cdot \text {LogLik} + 2 \cdot k, \qquad \text {BIC} = -2 \cdot \text {LogLik} + \log (n) \cdot k, \end{aligned}$$

where $\text {LogLik}$ is the maximized log-likelihood of the model, *k* is the number of estimated parameters, and *n* is the sample size.

Decreased values of AIC, BIC, and RMSE improve the model performance. Lower KS statistics suggest a better alignment between the fitted distributions and the empirical data. The selected model must meet these criteria to ensure accurate predictions of lung survival.

**Table 3:** Model comparison for lung survival data. Estimates (with standard errors) and model selection criteria ($-2$logL, AIC, BIC, RMSE, KS, p-value) are shown for exponential, Weibull, LTED-Exp, and LTED-Weib models.

Model	$\theta \hbox {(SE)}$	$\alpha$(SE)	$\beta$(SE)	$\lambda$(SE)	$\tau$(SE)	$-2$logL	AIC	BIC	RMSE	KS	p-value
Exponential	0.85(0.08)	–	–	–	–	281.94	283.94	284.17	0.137	0.152	< 0.001
Weibull	–	1.35(0.10)	1.16(0.08)	–	–	267.46	271.46	271.91	0.070	0.157	< 0.001
LTED-Exp	0.18(0.04)	–	–	1.40(0.12)	9.96(1.48)	267.94	273.59	274.62	0.067	0.151	< 0.001
LTED-Weib	–	0.27(0.00)	1.58(0.01)	0.60(0.03)	0.10(0.01)	262.80	270.80	271.70	0.110	0.155	< 0.001

As shown in Table 3, the LTED-Weib model offers the best overall fit to the data based on the AIC and BIC criteria, while the Weibull model closely follows in performance. The LTED-Exp model shows a lower KS statistic, which indicates a small discrepancy between the empirical and fitted distributions. However, the AIC and BIC values for the LTED-Exp model are higher than those for both the LTED-Weib and Weibull models. This result indicates that, when evaluating overall likelihood-based criteria, the LTED-Exp model is less favored. The exponential model shows the poorest fit compared to the other candidates. In addition, the exponential model displays the higher RMSE, whereas the Weibull and LTED-based models show reduced RMSE, but the LTED-Exp model achieves superior performance. It is important to note that the KS test p-values for all models are less than 0.001, indicating that no model perfectly fits the empirical data.

**Figure 7:**
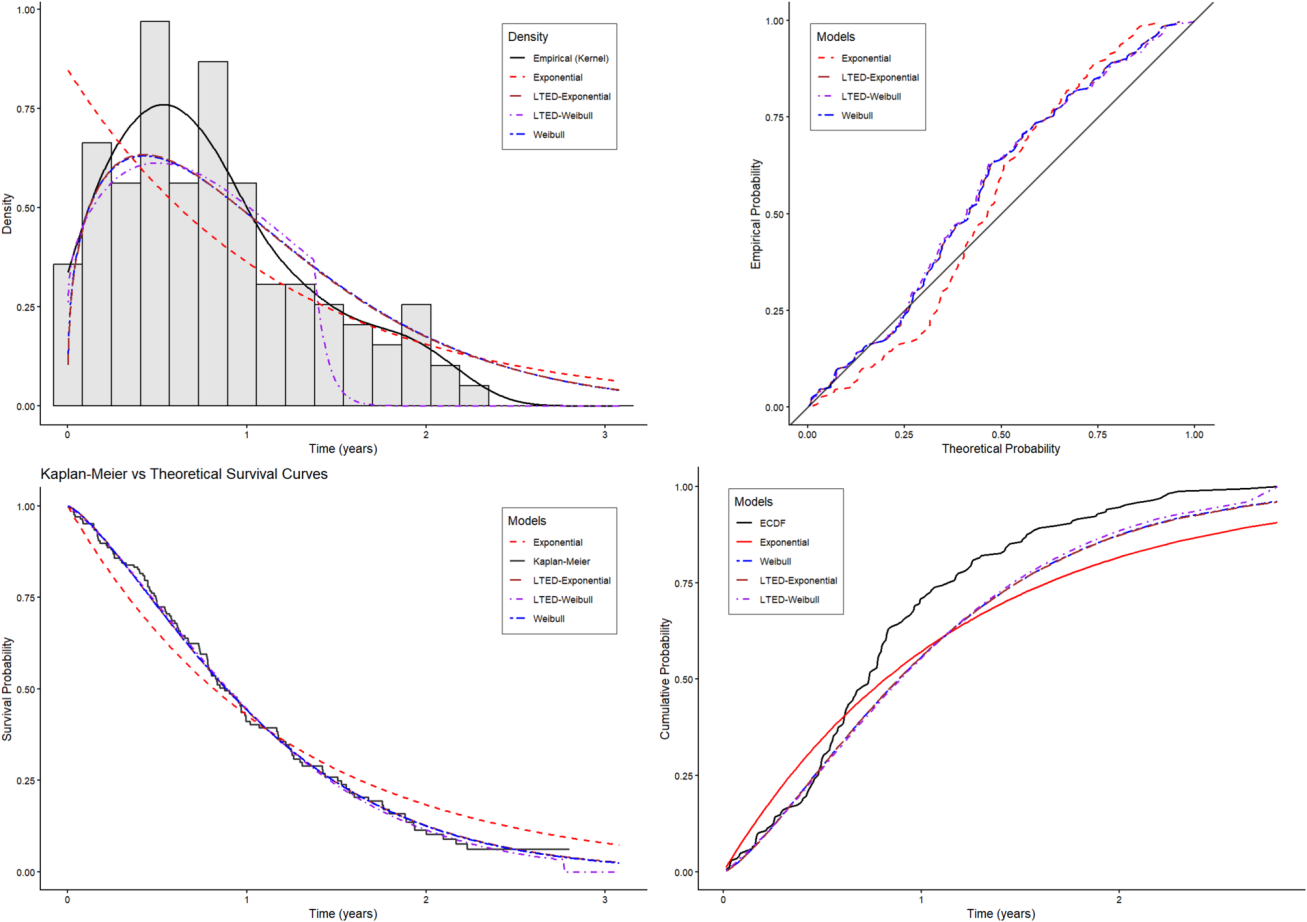
Comparison of empirical lung survival data with fitted models. Top row: histogram with fitted density and expected probabilities. Bottom row: Kaplan-Meier survival curves with theoretical and empirical cumulative distributions for exponential, Weibull, LTED-Exp, and LTED-Weib models.

Figure 7 presents a comparison between empirical survival data and the fitted models. Both LTED variants closely follow the empirical shape and capture certain tail behaviors more effectively than the exponential model, although the differences among the models are subtle. The LTED extensions offer a flexible methodology for modeling lung survival data. The performance of the models should be viewed as competitive rather than definitively superior.

## Discussion and concluding remarks

This paper introduced the LTED, a new flexible distribution family that extends the classical Exp-*F* family through a nested two-stage exponentiation mechanism. This extension allows simple baseline distributions to model highly skewed or heavy-tailed data while retaining closed-form quantiles and explicit moment expressions.

Simulation studies demonstrate that for the LTED-Exp, MLE and WLS provide the most reliable parameter estimates across a range of sample sizes. For the LTED-Weib, estimator performance varies by parameter and sample size, but all methods improve with increasing *n*. These results provide practical guidance for the selection of estimation techniques when applying LTED in real-world studies.

Applying LTED to lung survival data illustrates its practical utility in real-life scenarios. The LTED-Weib model fits better than existing exponential and Weibull models because it captures both central tendencies and tail behavior more accurately. The LTED is not merely a theoretical extension, it also provides tangible improvements in data modeling.

The LTED improves distribution of families to further advance lifetime statistical modeling. This expansion enables more effective modeling of skewness, tail behavior, and hazard rates. It offers closed-form and tractable properties that simplify inference, simulation, and practical applications. Additionally, the framework is adaptable to various baseline distributions, increasing its utility in reliability, survival, and risk analysis.

Future research may focus on improving the LTED by integrating Bayesian estimation, regression modeling, and multivariate frameworks. This investigation would highlight its potential as a tool across multiple areas of statistical application.

## Data Availability

The data supporting the findings of this study can be acquired from the corresponding author upon request.
